# Static magnetization switching in an artificial antiferromagnetic multilayer driven by a voltage-controlled magnetic anisotropy effect

**DOI:** 10.1038/s41563-026-02575-w

**Published:** 2026-04-03

**Authors:** Hiroyasu Nakayama, Takayuki Nozaki, Toshiki Yamaji, Tomohiro Nozaki, Hiroshi Imamura, Shinji Yuasa

**Affiliations:** 1 National Institute of Advanced Industrial Science and Technology, Research Institute for Hybrid Functional Integration, Tsukuba, Japan; 2 PRESTO, Japan Science and Technology Agency, Kawaguchi, Japan; 3 National Institute of Advanced Industrial Science and Technology, Research Strategy Headquarters, Tsukuba, Japan

**Keywords:** Electronic devices, Information storage, Magnetic properties and materials, Electronic properties and materials, Electrical and electronic engineering

## Abstract

Voltage-induced magnetization switching based on the voltage-controlled magnetic anisotropy (VCMA) effect is expected to be the ultimate low-power-consumption writing method for spintronic devices such as non-volatile magnetoresistive random-access memory. However, for conventional VCMA-driven dynamic magnetization switching, in which sub-nanosecond voltage pulses induce bidirectional switching by inducing a half precession of magnetization, even a small variation in the pulse widths of the order of several picoseconds can cause switching failure. This has become a major obstacle for developing voltage-controlled magnetoresistive random-access memory. Here we report VCMA-driven static magnetization switching by exploiting an artificial antiferromagnetic trilayer structure with interlayer exchange coupling. By applying bipolar voltages to the antiferromagnetic structure, we can demonstrate repeatable bidirectional switching. Unlike conventional dynamic switching, VCMA-driven static magnetization switching is induced in a wide range of pulse widths. This unconventional writing method is expected to be a key for developing various ultralow-power spintronic devices.

## Abstract (web summary)

For conventional voltage-controlled magnetic anisotropy (VCMA) switching, a small variation in the pulse widths of the order of several picoseconds can cause switching failure. Now, VCMA-driven static magnetization switching is demonstrated, which is largely insensitive to pulse widths within certain thresholds.

## Main

**Fig. 1:**
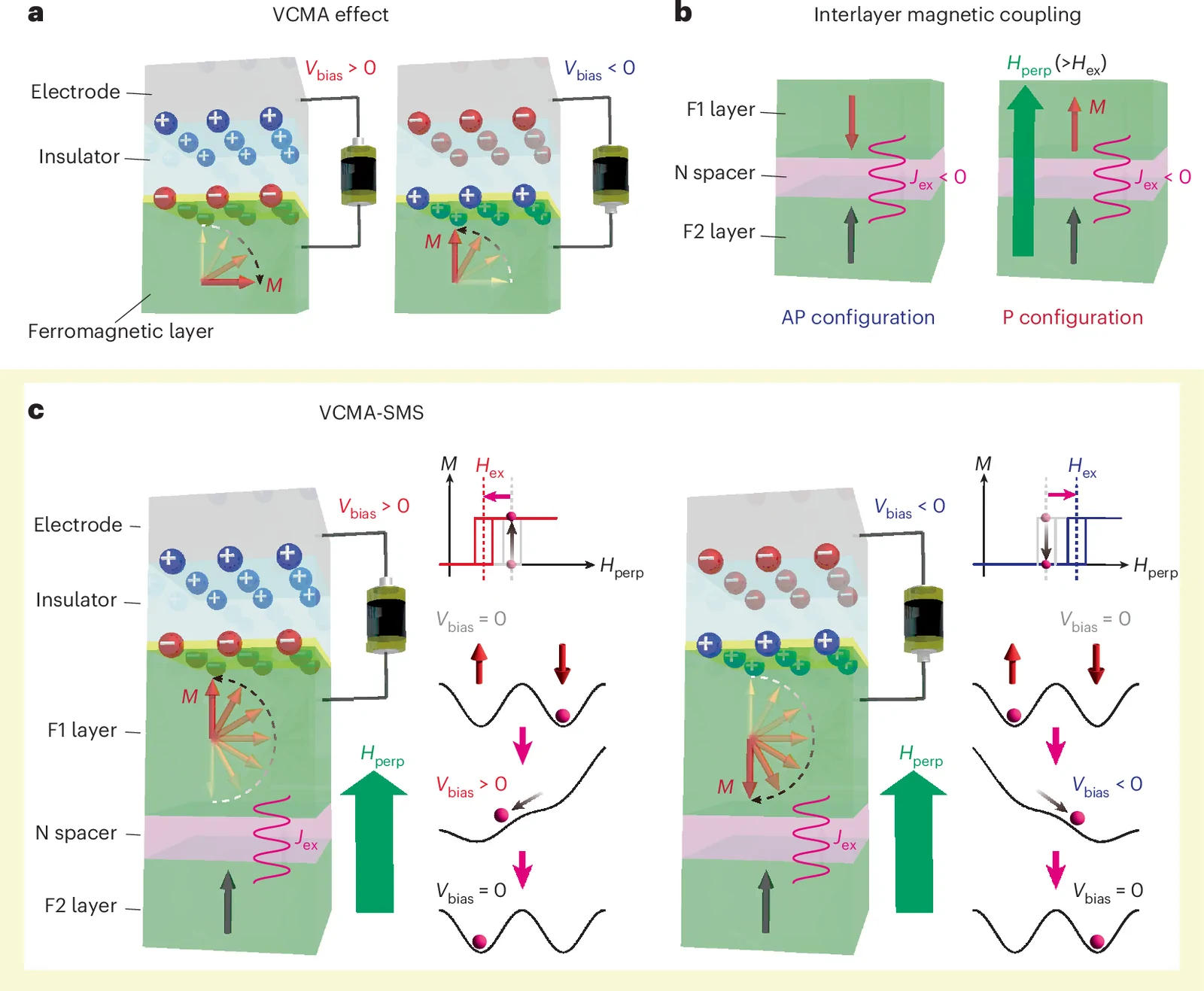
Concept of VCMA and VCMA-SMS. **a**, Schematic of the VCMA effect. When a bias voltage *V*_bias_ is applied, charge accumulates at the interface of the ferromagnetic layer, resulting in a change in magnetic anisotropy. **b**, Schematic of artificial (synthetic) antiferromagnetic trilayer with the IEC. When the IEC energy *J*_ex_ is negative, the magnetization configuration becomes AP. Conversely, when a sufficiently strong magnetic field is applied, or when *J*_ex_ is positive, the magnetization configuration becomes P. **c**, Schematic of the VCMA-SMS in a ferromagnet (F2)/non-magnetic spacer (N)/ferromagnet (F1) multilayer with interlayer coupling. Even when *J*_ex_ is constant, the effective exchange field *H*_ex_ decreases (increases) with a decrease (increase) in PMA of the F1 layer induced by the VCMA effect. Under certain static magnetic field conditions, the application of a positive (negative) bias voltage *V*_bias_ modifies the magnetic energy potential landscape, resulting in the switching of magnetization configuration from AP to P (P to AP).

The electrical control of magnetization has been one of the central issues in the field of spintronics for more than two decades since it is a basis of energy efficient magnetization switching (writing) in magnetic recording and non-volatile magnetic memory such as magnetoresistive random-access memory (MRAM)^1^. In current mainstream MRAMs, called spin-transfer torque (STT) MRAM, the magnetization of nanosized magnetic tunnel junctions (MTJs) is reversed (switched) by a pulse of electric current, which inevitably yields Joule loss of several tens of femtojoules per bit. To avoid such Joule loss, the voltage control of magnetization has been intensively studied^2–20^. The use of the voltage-controlled magnetic anisotropy (VCMA) effect is one of the most promising approaches^2,3,5,12,16,17,19^. The VCMA effect takes place at the interface between a ferromagnetic metal and non-magnetic insulator (tunnel barrier), where an application of electric field via the insulator changes the interfacial perpendicular magnetic anisotropy (PMA) energy (Fig. 1a). The VCMA effect alone can rotate the magnetization between in-plane and out-of-plane directions but cannot reverse the magnetization. When an ultrashort voltage pulse is applied under a static magnetic field, on the other hand, the VCMA effect can reverse the magnetization as a result of the precessional motion of magnetization^8,10^. An application of voltage with a rise time shorter than several tens of picoseconds induces a large-angle precession of magnetization around the static field axis. By removing the voltage when the magnetization is rotated by 180° (that is, half precession), the magnetization stops, resulting in magnetization reversal. This type of magnetization reversal is called dynamic switching^8,10^. The dynamic switching induced by purely electronic VCMA effect is free from chemical reactions and the displacement of ions^4,6,7,13^.

The energy consumption of VCMA-driven dynamic switching can be ultralow, down to several femtojoules or below^21,22^, because the switching is not induced by current but by voltage. However, dynamic switching has a fundamental difficulty that even small deviations in the voltage pulse width from the optimal width for half precession causes switching failure^23^. Therefore, the pulse width should be precisely controlled typically in the order of a few hundred picoseconds^23^. Such a precise control of the pulse width is difficult in large-scale integrated circuits with circuit delays. Moreover, the optimal pulse width varies from MTJ to MTJ, which makes the operation of large-scale memory even more difficult. It should be noted that such a precise control of the pulse width is not necessary in STT MRAM. This is because reliable switching with a reasonably low current density is possible when the current pulse is longer than about 10 ns (ref. ^1^). Ideal voltage-induced magnetization switching for MRAM should be pulse-width insensitive.

One approach for such ideal voltage-induced switching is to modulate the interlayer exchange coupling (IEC) by the VCMA effect. The IEC is a long-range quantum mechanical interaction between two ferromagnetic layers (F1 and F2) separated by a nanometre-thick non-magnetic spacer layer (N)^24–27^. Depending on the spacer layer thickness, the F1/N/F2 trilayer exhibits antiparallel (AP) or parallel (P) magnetization configurations (Fig. 1b) due to the antiferromagnetic or ferromagnetic IEC. The trilayer with a strong negative (antiferromagnetic) IEC is called an artificial antiferromagnet or synthetic antiferromagnet. The magnetic field at which the magnetization configuration switches from AP to P is called the exchange field^28^. Recently, the voltage-controlled exchange coupling (VCEC) effect has been reported using ionic liquids, ferromagnetic semiconductors and ultrathin ferromagnetic layers^14,15,18,29–31^. By using the VCEC effect, however, high-speed switching is difficult to achieve when the switching is accompanied with the displacement or migration of atoms^14,15,29^. Moreover, the extremely small Thomas–Fermi screening length in metallic ferromagnets substantially limits the efficiency of VCEC^18,31^.

Here we propose a new method to achieve voltage-controlled IEC and magnetization switching by introducing the VCMA effect in an artificial antiferromagnetic multilayer structure (Fig. 1c), which we call VCMA-driven static magnetization switching (VCMA-SMS). We show that the effective exchange field *H*_ex_ is largely modulated by the PMA, which is effectively controlled by the VCMA effect. By exploiting the voltage-controlled *H*_ex_, we demonstrate bidirectional magnetization switching by means of static bipolar voltages. Moreover, we experimentally demonstrate the high-speed operation of VCMA-SMS by using short voltage pulses.

## Results

### Engineering *H*_ex_ by using heavy metals

**Fig. 2:**
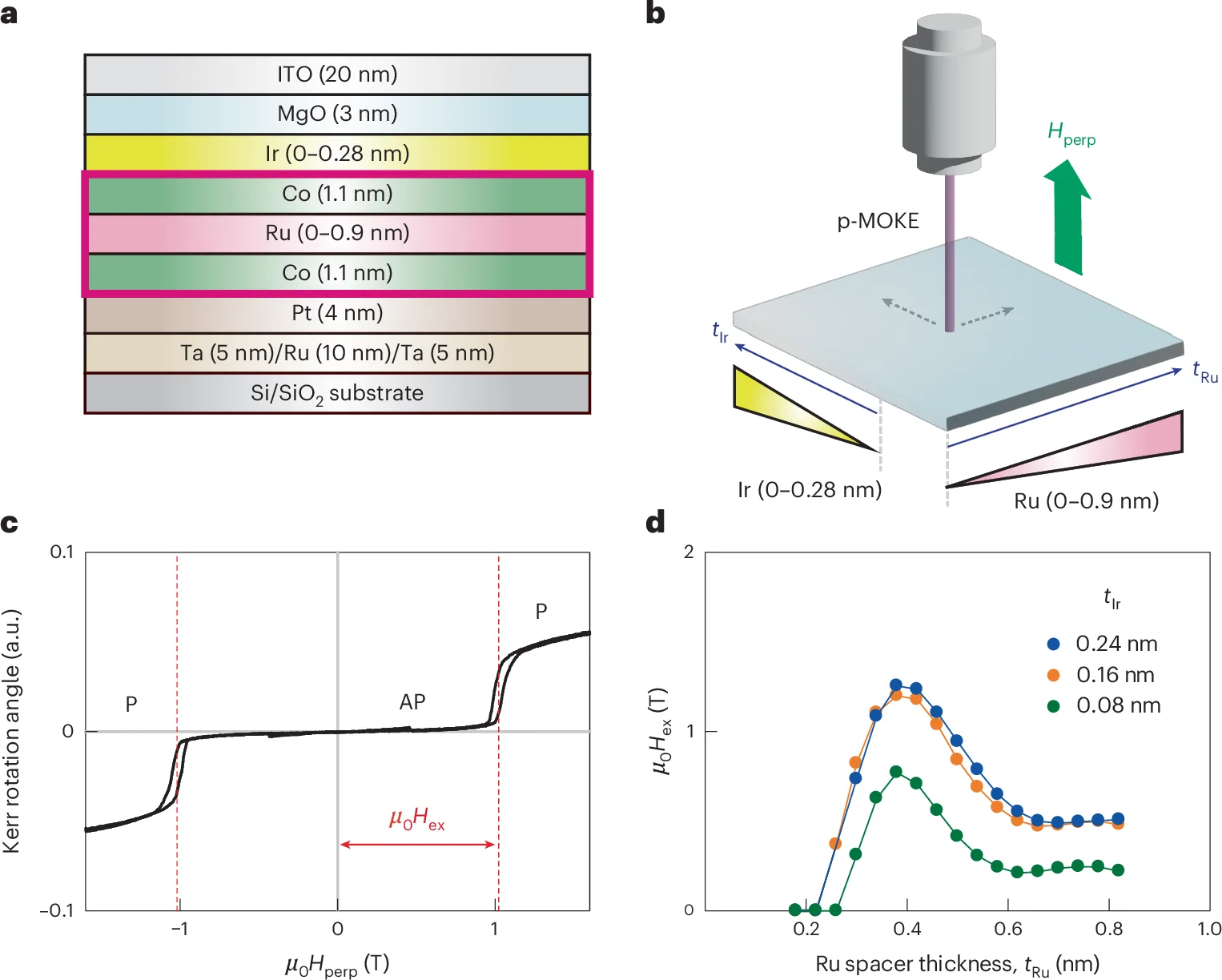
IEC in Co/Ru/Co trilayer structure. **a**, Schematic of the multilayer structure for IEC measurements. **b**, Schematic of the measurement configuration for p-MOKE. **c**, Typical p-MOKE magnetization curve of Co/Ru/Co trilayer, where Ru thickness (*t*_Ru_) is 0.35 nm and Ir thickness (*t*_Ir_) is 0.11 nm. Switching from the AP to P configuration takes place at the exchange field (*H*_ex_) of the IEC. **d**, *μ*_0_*H*_ex_ plotted as a function of *t*_Ru_ for various *t*_Ir_ values. Source data

We first studied the Co/Ru/Co trilayer structure with IEC and investigated the influence of the PMA of the top Co layer on the magnetization process. To tune the PMA, we deposited ultrathin Ir (0–0.28 nm)/MgO on the top Co layer. In our previous studies on the Co/ultrathin Ir (0–0.1 nm)/MgO structure, PMA of the Co layer is largely enhanced by increasing the thickness of the Ir layer^32,33^. Thus, we prepared substrate/seed/buffer layers/Co (1.1 nm)/Ru spacer (0–0.9 nm)/Co (1.1 nm)/ultrathin Ir (0–0.28 nm)/MgO (3 nm)/indium tin oxide (20 nm) polycrystalline film on a thermally oxidized silicon substrate (Fig. 2a and Methods). The magnetic properties of the multilayer films were evaluated by the polar magneto-optical Kerr effect (p-MOKE; Fig. 2b and Supplementary Note 1).

Figure 2c shows a typical p-MOKE magnetization curve for Co/Ru/Co with *t*_Ru_ = 0.35 nm and *t*_Ir_ = 0.11 nm, which exhibits an antiferromagnetic IEC, measured under a magnetic field applied perpendicular to the film plane (*H*_perp_). When |*H*_perp_| > *H*_ex_ is applied, the magnetic state switches from the AP to P configuration. The magnetization curve is discussed in more detail in Supplementary Note 2. Because the switching of magnetization configuration is accompanied with a hysteresis loop, we define the centre of the hysteresis loop as *H*_ex_. Hereafter, we focus on positive magnetic fields for simplicity because the magnetization process is symmetric with respect to *H*_perp_. We investigated the Ru thickness (*t*_Ru_) dependence of *H*_ex_ in Co/Ru/Co because the magnitude of *H*_ex_ is known to exhibit oscillatory behaviour with respect to *t*_Ru_ (ref. ^26^). In Fig. 2d, *H*_ex_ is plotted as a function of *t*_Ru_. Here the first peak appears at *t*_Ru_ ≈ 0.4 nm, which is consistent with our previous work on Co-based multilayers and Ru spacer^27^. An increase in *t*_Ir_, which causes an increase in the PMA of top Co layer, tends to increase *H*_ex_. Note that the data for *t*_Ir_ = 0 nm is not shown because the magnetization of Co is aligned in the film plane. *H*_ex_ increases with respect to *t*_Ir_ and tends to saturate when *t*_Ir _≥ 0.16 nm. These results suggest that *t*_Ir_ < 0.16 nm will be suitable for controlling *H*_ex_ by voltage.

The maximum value of the effective exchange coupling energy *J*_ex_ = *μ*_0_*H*_ex_*M*_S_*t*_Co_* is evaluated to be 1.8 mJ m^−2^, where *μ*_0_ is the permeability of vacuum, *t*_Co_*(≡*t*_Co_ − *t*_dead_) is the effective thickness of the Co layers, supposing that the saturation magnetization *M*_S_ of Co is 1,417 kA m^−1^, the thickness of Co layers (*t*_Co_) is 1.1 nm, and the thickness of the magnetically dead layer (*t*_dead_) is 0.1 nm (refs. ^33,34^). This *J*_ex_ value is comparable with the value reported in our previous work^27^.

### Voltage control of *H*_ex_

**Fig. 3:**
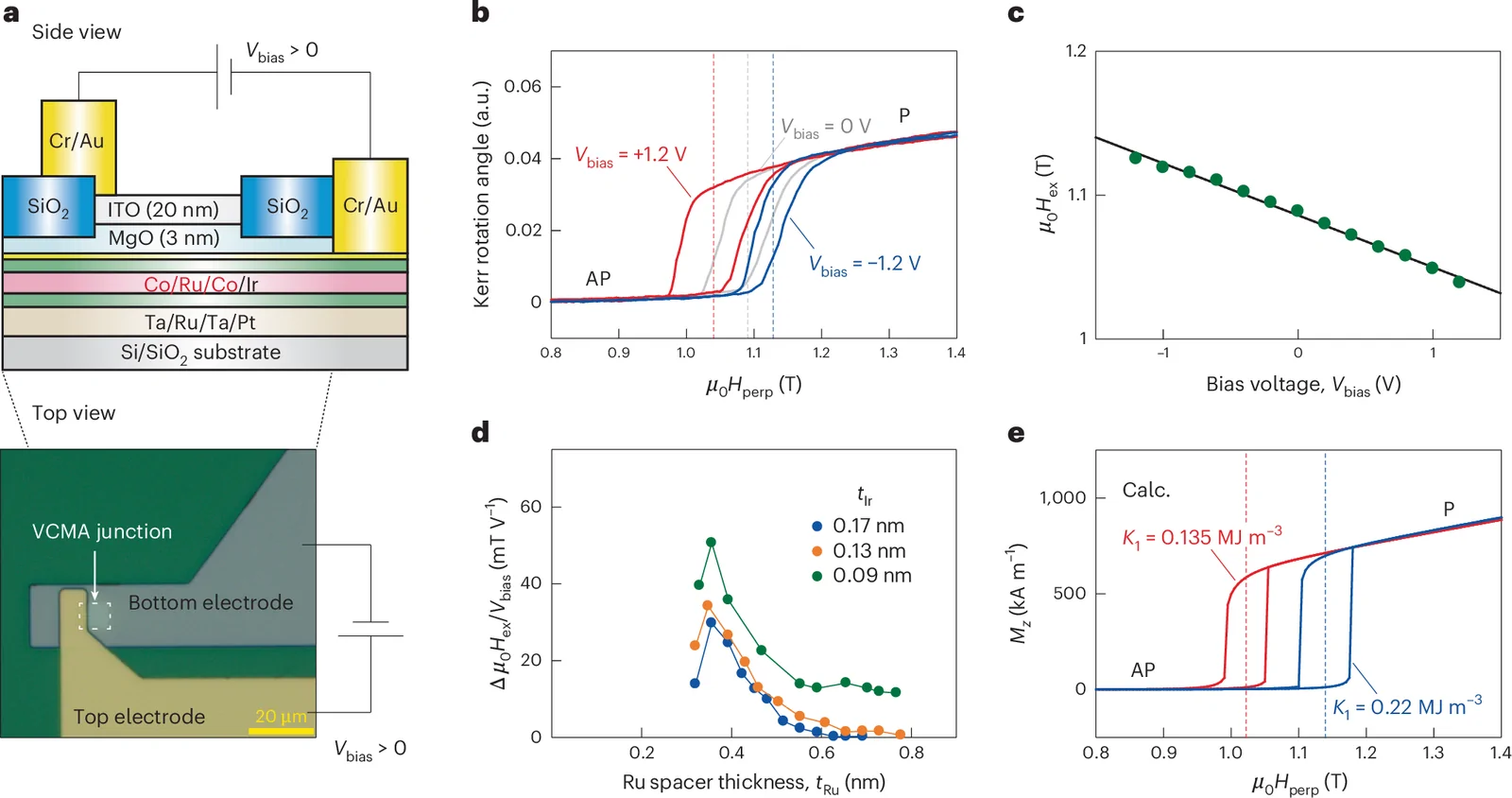
Voltage-controlled effective *H*_ex_ based on VCMA effect. **a**, Schematic and photograph of the device structure for studying the VCMA-driven phenomena. **b**, Bias-voltage-dependent p-MOKE magnetization curves in the vicinity of *μ*_0_*H*_ex_, where *t*_Ru_ = 0.34 nm and *t*_Ir_ = 0.11 nm. **c**, *V*_bias_ dependence of *μ*_0_*H*_ex_. **d**, Efficiency of the voltage control of *H*_ex_, Δ*μ*_0_*H*_ex_/*V*_bias_, plotted as function of *t*_Ru_. **e**, Calculated magnetization curves. Although *J* is constant, the effective *H*_ex_ depends on the PMA energy of the top Co layer (*K*_1_). Source data

To study VCMA-driven phenomena, we fabricated the multilayer film shown in Fig. 2a into a device structure schematically illustrated in Fig. 3a by using typical microfabrication techniques. The lateral size of the VCMA junction in Fig. 3a is 10 × 8 μm. Figure 3b shows the typical p-MOKE magnetization curves of Co/Ru/Co trilayer with *t*_Ru_ = 0.34 nm and *t*_Ir_ = 0.11 nm in the vicinity of *μ*_0_*H*_ex_ for applied bias voltages with different polarities. When a negative (positive) voltage is applied to the device, PMA and *H*_ex_ increase (decrease) due to the VCMA effect. These behaviours are consistent with the results shown in Fig. 2. As shown in Fig. 3c, *H*_ex_ linearly decreases with the applied bias voltage (*V*_bias_), which is also consistent with the sign of the VCMA effect. The negative slope shown in Fig. 3c (–Δ*μ*_0_*H*_ex_/*V*_bias_) is the efficiency of the voltage control of *H*_ex_.

To efficiently control *H*_ex_ by means of voltage, we optimized the multilayer structure. Figure 3d plots the *t*_Ru_ dependence of Δ*μ*_0_*H*_ex_/*V*_bias_ for various *t*_Ir_ values. Large Δ*μ*_0_*H*_ex_/*V*_bias_ was obtained in the vicinity of the first peak of *H*_ex_ near *t*_Ru_ = 0.4 nm (Fig. 2d). The largest value of Δ*μ*_0_*H*_ex_/*V*_bias_ is about 52 mT V^−1^, which corresponds to the efficiency of the effective VCEC, defined as (Δ*J*_ex_/Δ*V*_bias_) × *t*_barrier_ = (Δ*μ*_0_*H*_ex_*M*_S_*t*_Co_*/Δ*V*_bias_) × *t*_barrier_, of −180 fJ V^−1^ m^−1^, where *t*_barrier_ is the thickness of the tunnel barrier. Surprisingly, this value is two orders of magnitude larger than the largest value reported in previous studies on the VCEC effect^31^, clearly suggesting that the VCMA effect is an effective way for tuning the effective IEC by voltage. It should be noted that the physical origins of the VCEC effect observed in previous studies^14,15,18,29,31^ are different from the VCMA effect. The term VCEC is not strictly correct in this study because the IEC is not directly controlled by voltage but is effectively tuned by the VCMA effect.

To understand the observed effective voltage-controlled IEC in more detail, we conducted macrospin calculations on the magnetization curves of an artificial antiferromagnetic trilayer (Supplementary Note 5). Figure 3e shows the magnetization curves of the artificial antiferromagnetic trilayer with different PMA energies of the top Co layer (*K*_1_). Here the PMA energy of the bottom Co layer (*K*_2_), interlayer exchange coupling constant (*J*), saturation magnetization of Co layers and thickness of Co layers are fixed at 0.185 × 10^6^ J m^−3^, −3.25 × 10^−3^ J m^−2^, 1,400 kA m^−1^ and 1.0 nm, respectively. The Gilbert damping constants were set to 1.0 to reduce the computation time. The tendency of the experimental results that *H*_ex_ increases (decreases) with increasing (decreasing) PMA of the top Co layer (Fig. 2d) is reproduced by the calculations. Even though the exchange coupling constant *J* remains unchanged, the effective *H*_ex_ is tuned by the PMA of the top Co layer. The important point here is that the effective *H*_ex_ can be manipulated solely by voltage. Because *H*_ex_ largely depends on the applied voltage, we can expect voltage-induced bidirectional magnetization switching under a fixed magnetic field. It should be noted that the macrospin model cannot account for the spatial inhomogeneity of magnetization or domain nucleation. Therefore, the model is not applicable to systems exhibiting multidomain formation. Large-scale micromagnetic simulations would be required for a fully quantitative comparison with experiments, as they are essential for accurately capturing the non-uniform magnetization dynamics observed in real devices.

We also studied junctions with the opposite sign of the VCMA effect (Supplementary Note 3). By changing the materials and stacking structure of the multilayer samples, we inverted the sign of the VCMA effect and observed the inversion of the slope of *μ*_0_*H*_ex_–*V*_bias_ relationship (Supplementary Fig. 3c). This is additional evidence that the origin of the voltage-controlled effective *H*_ex_ and *J*_ex_ is the VCMA effect.

### VCMA-SMS

**Fig. 4:**
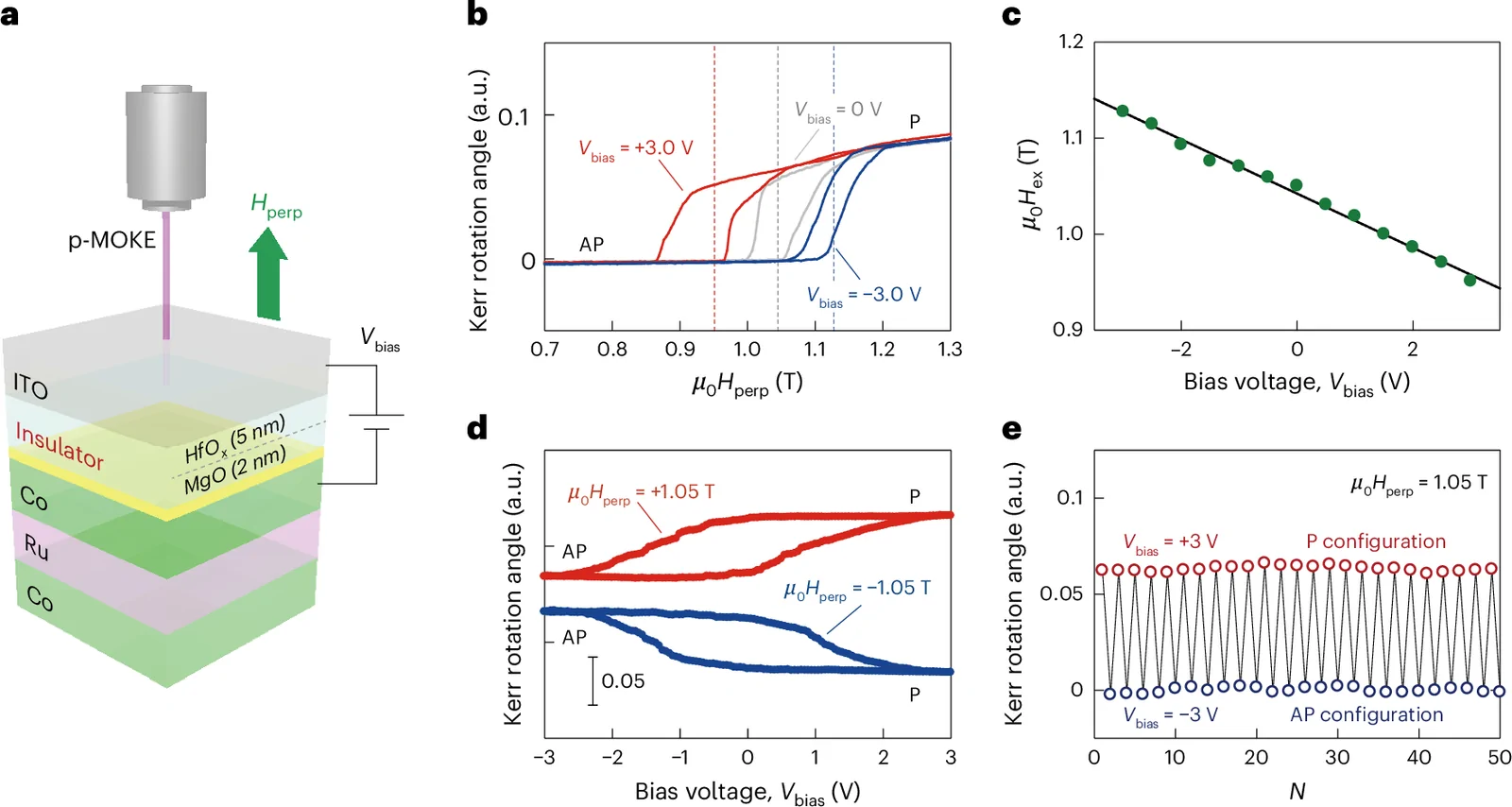
Experimental demonstration of VCMA-SMS. **a**, Schematic of the experimental configuration with p-MOKE measurement. To enhance the VCMA, we used a hybrid insulating barrier with a high-*k* material HfO_*x*_. **b**, p-MOKE hysteresis curves of the multilayer device, where *t*_Ru_ and *t*_Ir_ are 0.39 nm and 0.10 nm, respectively. **c**, *V*_bias_ dependence of *μ*_0_*H*_ex_. **d**, *V*_bias_ dependence of p-MOKE signal under fixed *μ*_0_*H*_perp_ with different polarities. **e**, Change in p-MOKE signal by alternately applying positive (*N* = 1, 3, 5…) and negative (*N* = 2, 4, 6…) *V*_bias_. Source data

To effectively demonstrate VCMA-SMS, we first tried to enhance the VCMA and the voltage-controlled *H*_ex_ by using MgO (2 nm)/HfO_*x*_ (5 nm) as the insulating tunnel barrier layer which has a larger dielectric constant (Fig. 4a and Methods)^35^. Figure 4b shows the magnetization curves in the vicinity of *μ*_0_*H*_ex_, where *t*_Ru_ = 0.39 nm and *t*_Ir_ = 0.10 nm. The *V*_bias_ dependence of *μ*_0_*H*_ex_ (Fig. 4c) corresponds to a VCEC efficiency of −310 fJ V^−1^ m^−1^, which is substantially higher than that of the samples in Fig. 3 (−180 fJ V^−1^ m^−1^). The enhanced voltage-controlled *H*_ex_ and *J*_ex_ are attributed to the larger VCMA effect. Compared with the voltage-dependent hysteresis loops shown in Fig. 3b, the hysteresis loops for positive and negative voltages shown in Fig. 4b are clearly separated from each other. This indicates that the magnetization configuration can be switched solely by voltage when the magnetic field is fixed at the centre of these hysteresis loops. We also fabricated devices with the same structure and nominal film thickness on different substrates and evaluated the variation in *μ*_0_*H*_ex_. Even among different deposition batches, the device-to-device variation in *μ*_0_*H*_ex_ remains small, with a standard deviation of approximately 4 mT. Such a variation in *μ*_0_*H*_ex_ does not pose a serious problem for the write operation in the present scheme.

Figure 4d shows the experimental results of purely voltage-induced magnetization switching under a fixed magnetic field. A clear hysteresis loop was observed at *μ*_0_*H*_perp_ = +1.05 T. At *V*_bias_ = 0, the magnetization configuration exhibits bistable states corresponding to the AP and P configurations. This is a successful demonstration of VCMA-SMS. A similar VCMA-SMS takes place at *μ*_0_*H*_perp_ = –1.05 T. We repeated VCMA-SMS to confirm its reliability. Positive and negative voltages (±3 V) were alternately applied by sweeping the voltage. To observe purely voltage-induced effects, here we used a thick insulating layer with a thickness of 7 nm, which leads to a high electrical resistance (~1 GΩ). The maximum charge current density is less than 38 A m^−2^, which is several orders of magnitudes smaller than that for STT switching. Figure 4e shows the p-MOKE signal for Co/Ru/Co at positive (*N* = 1, 3, 5…) and negative (*N* = 2, 4, 6…) voltages, where *N* represents the cycle index. Repeatable switching events were confirmed with d.c. voltages. In Fig. 4e, the p-MOKE signal for AP and P configurations slightly varies at each switching event. This suggests that the junction sample with a large lateral size (10 × 8 μm) does not have a perfectly single magnetic domain. Such variation will be eliminated simply by shrinking the sample area to approach a single-domain state.

### VCMA-SMS with short voltage pulses

Next, we demonstrate VCMA-SMS by applying short voltage pulses to the same sample and prove its applicability to high-speed memory devices. Here we performed p-MOKE measurements after applying voltage pulses with pulse widths of tens of nanoseconds. It should be noted that voltage pulses in this width range can easily be controlled in large-scale memory chips with circuit delay. Note also that voltage pulses with such widths cannot induce the conventional VCMA-induced dynamic switching^8,10^. To apply short voltage pulses, we used an arbitrary function generator. Before the measurements, a magnetic field of +1.8 T was applied to initialize the magnetic configuration at the P state. We measured the p-MOKE signals before and after applying voltage pulses at fixed *H*_perp_ of +1.05 T.

**Fig. 5:**
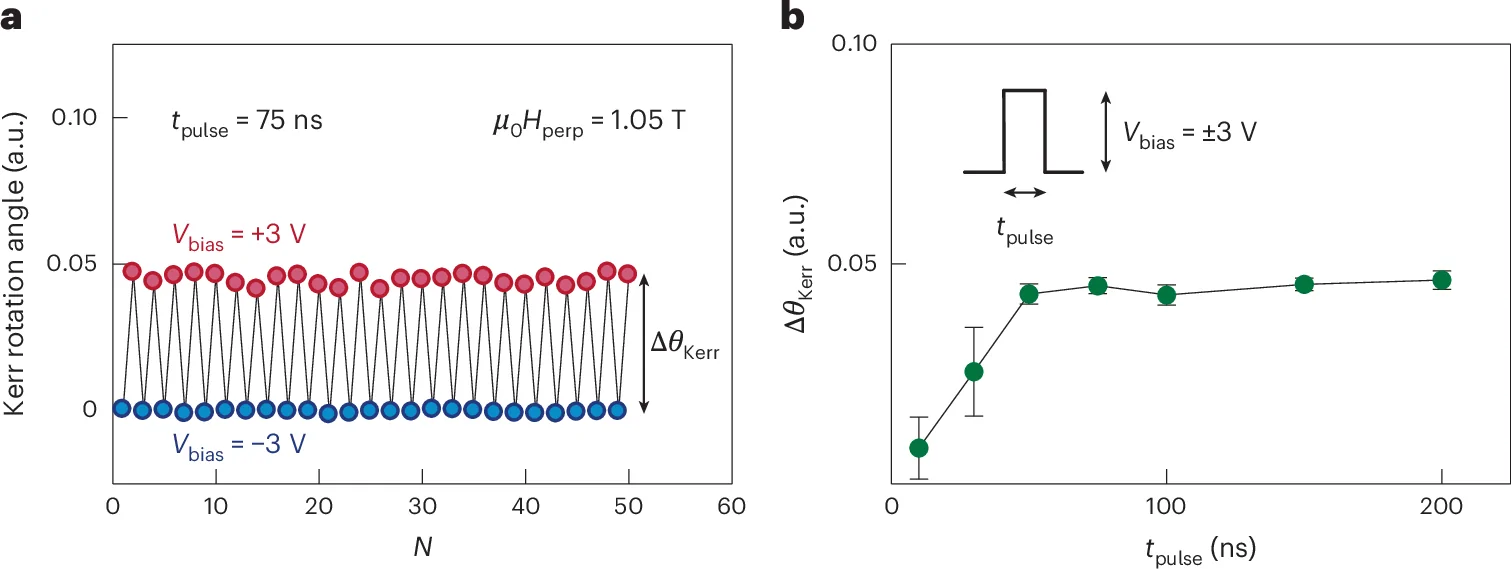
VCMA-SMS by voltage pulse. **a**, Change in the p-MOKE signal by alternately applying negative (*N* = 1, 3, 5…) and positive (*N* = 2, 4, 6…) voltage pulses with a pulse width of 75 ns. **b**, Change in p-MOKE signal between the AP and P states (Δ*θ*_Kerr_) due to VCMA-SMS as a function of the voltage pulse width (*t*_pulse_). Here the plotted values represent the mean ± s.d., both calculated from 50 measurements performed on the same sample shown in **a**. Source data

We first show the experimental result for the pulse width of 75 ns. Figure 5a shows the p-MOKE signals after the application of negative (*N* = 1, 3, 5…) and positive (*N* = 2, 4, 6…) voltage pulses. Repeatable switching events were demonstrated with the voltage pulse as in the case of d.c. voltage. It should be noted that the p-MOKE signal for the P state (Fig. 5a) is slightly smaller than that in Fig. 4e. This is mainly because p-MOKE shown in Fig. 5a was measured after the voltage was removed, whereas p-MOKE shown in Fig. 4e was measured under the applied voltage.

Next, we conducted similar experiments with different pulse widths to investigate how fast the magnetization can be switched. Figure 5b plots the average change in the p-MOKE signal between the AP and P states (Δ*θ*_Kerr_) due to VCMA-SMS as a function of the voltage pulse width (*t*_pulse_). Reliable VCMA-SMS was observed with *t*_pulse_ down to 50 ns, below which switching was imperfect. Reliable pulse-voltage-induced VCMA-SMS has been experimentally demonstrated in pulse widths ranging from 50 ns to ∞ (d.c. voltage).

Although VCMA-SMS with a pulse width of 50 ns is potentially applicable to voltage-controlled MRAM for embedded non-volatile memory, the optimum pulse width in terms of operation speed, circuit design and power consumption of non-volatile working memory is about 5–10 ns (ref. ^1^). Note that a pulse width of 5–10 ns is too long to induce the conventional VCMA-driven dynamic switching^8,10^. Here we discuss whether VCMA-SMS can be induced in this pulse width range. We should first emphasize that the observed VCMA-SMS with the pulse width of 50 ns is rather surprising because the sample is large (10 × 8-μm^2^-sized junction) and, therefore, is not a single magnetic domain. In the case of STT switching and VCMA-driven dynamic switching, such large junctions do not exhibit reliable switching because a single-domain state is essential for these types of switching. As the junction size shrinks below 100 nm and the magnetic state approaches a single-domain state, the junction generally shows more reliable and faster switching. To theoretically confirm this point, we performed a macrospin simulation of VCMA-SMS by assuming a single-domain state. We found that the minimum pulse width for inducing VCMA-SMS, *t*_sw_, becomes shorter as the Gilbert damping of the ferromagnetic layer increases. This relationship between magnetic damping and switching speed is similar to that for magnetic-field-induced switching used in magnetic recording devices such as hard disc drives, in which the magnetic media with high damping materials are used for faster writing. Our macrospin simulation with a damping constant of *α* = 0.05–0.1, which is reasonable for ferromagnetic alloys based on Fe–Pt and Co–Pt, showed that *t*_sw_ can be shorter than about 1 ns. The macrospin simulation assumes a single-domain state. A single-domain state can be realized in MTJs with perpendicular magnetization when the junction diameter is smaller than 100 nm. By using such small junctions, we can expect reliable VCMA-SMS in the wide range of pulse widths from 1 ns to d.c. voltage.

## Discussions

### Remaining challenges for applying VCMA-SMS to non-volatile memory

Last, we discuss the remaining major challenges for applying VCMA-SMS to voltage-controlled MRAM. The reliable VCMA-SMS in a wide range of pulse widths can solve the major problem of conventional voltage-induced switching in which the optimum pulse width is too short to be precisely controlled. However, some technical issues remain unsolved. First, VCMA-SMS requires a static magnetic field of about 1 T, which is too high to be applied in solid-state devices. Second, the VCMA efficiency and PMA should be enhanced for higher memory density. To reduce the required static magnetic field, it is effective to enhance the VCMA efficiency. This is because a higher VCMA efficiency results in a larger Δ*H*_ex_, allowing magnetization reversal with a smaller *H*_ex_ value. Although the VCMA efficiency of polycrystalline MTJs has been shown to be typically about 100 fJ V^−1^ m^−1^, an efficiency of up to about 400 fJ V^−1^ m^−1^ has been achieved in epitaxial MTJs. The VCMA efficiency of several hundred femtojoules per volt–metre can reduce the required magnetic field to the order of 0.1 T, which can be applied in solid-state devices by using the stray magnetic field from the pinned layers in junctions smaller than ~100 nm and/or by placing magnetic dots above MTJs. However, it should be noted that in devices influenced by stray fields, the width of hysteresis at around *H*_ex_ tends to increase in junctions smaller than ~100 nm (ref. ^36^). For utilizing the stray field from the pinned layer, materials with a larger VCMA effect may be necessary. It is, therefore, important to develop new materials to enhance both VCMA efficiency and PMA.

### Material design for VCMA-SMS

VCMA-SMS would have a clear advantage over dynamic switching and STT switching in that a variety of materials can be used. For dynamic switching and STT switching, a very low Gilbert damping (*α* ≤ 0.01) is required for reducing the switching energy and switching errors because dynamic switching and STT switching are based on the precession of magnetization, which is suppressed by damping^23,37^. Due to this requirement, we cannot use ferromagnetic alloys with heavy metals such as Pt and other 5*d* transition metals, which are known to be effective for enhancing the PMA and VCMA. In VCMA-SMS, on the other hand, a high Gilbert damping (*α* ≥ 0.05) is not a problem or could even be favourable because it is a phenomenon not based on the precession of magnetization. Therefore, VCMA-SMS will open a new way to utilize heavy metals in spintronics.

## Conclusions

We have demonstrated VCMA-SMS by using artificial antiferromagnetic multilayers. By using the VCMA effect, we changed the effective IEC of the artificial antiferromagnet by means of voltage and successfully achieved reliable VCMA-SMS under a static magnetic field. Unlike the conventional VCMA-driven dynamic switching, reliable VCMA-SMS has been experimentally demonstrated for a wide range of voltage pulse widths from 50 ns up to d.c. voltage. Further reduction in the pulse width down to 1 ns is theoretically possible by using small junctions with a single-domain state. VCMA-SMS is expected to be a key for developing various ultralow-power spintronic devices.

## Methods

### Sample preparation

The stacking structure of the polycrystalline film used for the experiments on voltage-controlled IEC is Ta (5 nm)/Ru (10 nm)/Ta (5 nm)/Pt (4 nm)/Co (1.1 nm)/Ru (0–0.9 nm)/Co (1.1 nm)/Ir (0–0.28 nm)/MgO (3 nm)/indium tin oxide (20 nm) deposited on a thermally oxidized silicon substrate, where the order of the layers is described from bottom to top. For the switching experiments, we replaced the MgO (3 nm) barrier with MgO (2 nm)/HfO_*x*_ (5 nm) to enhance the dielectric constant and VCMA effect^35^. The multilayer films were deposited at room temperature by a combination of sputtering deposition and molecular-beam epitaxy without breaking the vacuum. Most of the layers were deposited by magnetron sputtering, and only the MgO layers were deposited by electron-beam evaporation. To obtain IEC between the Co layers, we used Ru as a spacer layer. The Ir and Pt layers were used to increase the magnitude of PMA. The thicknesses of Ru (*t*_Ru_) and Ir (*t*_Ir_) were varied on each substrate by using a linear motion shutter during the deposition. For the samples with oxidation, which exhibits the positive VCMA effect, Ir was replaced by Pt. The oxidation process was conducted in pure oxygen gas at a pressure of 0.5 Pa for 3 min in an oxidation chamber directly connected to the sputtering deposition chamber^34^. For the top electrode, transparent indium tin oxide was used to evaluate the magnetic properties of Co layers by using p-MOKE. The samples for the switching experiments were annealed at 250 °C in a vacuum for 1 h to improve the quality of the insulating barrier layers^35^, whereas the samples for the experiments of the voltage-controlled IEC were not annealed. The VCMA devices were fabricated by conventional optical lithography and ion-milling processes. The lateral size of the tunnel junctions was 10 × 8 μm^2^.

### Measurements of magnetic properties

The magnetic properties of the multilayer films were evaluated by measuring p-MOKE, in which a semiconductor laser with a spot size of 1.5 μm was used. Because the artificial antiferromagnetic trilayer is very thin with respect to the penetration depth of the light, both top and bottom Co layers are sensed by p-MOKE, although the top Co layer contributes to the p-MOKE signal slightly more than the bottom Co layer. The change in the Kerr rotation angle was measured by applying an external magnetic field perpendicular to the film plane. For the experiments of voltage-controlled IEC and VCMA-SMS, the d.c. voltage was applied by a source measure unit, and the voltage pulse with the widths of 10–200 ns was applied by an arbitrary function generator. All the measurements were performed at room temperature.

## Online content

Any methods, additional references, Nature Portfolio reporting summaries, source data, extended data, supplementary information, acknowledgements, peer review information; details of author contributions and competing interests; and statements of data and code availability are available at https://doi.org/10.1038/s41563-026-02575-w.

## Supplementary information

Supplementary Information Supplementary Notes 1–5, Figs. 1–4 and references.

## Source data

Source Data Fig. 2 Source data for the plots in Fig. 2c,d.

Source Data Fig. 3 Source data for the plots in Fig. 3b–e.

Source Data Fig. 4 Source data for the plots in Fig. 4b–e.

Source Data Fig. 5 Source data for the plots in Fig. 5a,b.

## Data Availability

The data that support the findings of this study are available from the corresponding author upon request. Source data are provided with this paper.
