# Supplementary material for: Static magnetization switching in an artificial antiferromagnetic multilayer driven by a voltage-controlled magnetic anisotropy effect
Source: Nat Mater. 2026 Apr 3;25(10):1762–8. doi: 10.1038/s41563-026-02575-w (PMC13619440; doi:10.1038/s41563-026-02575-w)
Supplement: Supplementary file 1 [file 41563_2026_2575_MOESM1_ESM.pdf]

nature materials

Supplementary information

https://doi.org/10.1038/s41563-026-02575-w

Static magnetization switching in an artificial antiferromagnetic multilayer driven by a voltage-controlled magnetic anisotropy effect

In the format provided by the authors and unedited

Contents

Supplementary Note 1. Measurement setup for voltage-controlled H_{ex} and VCMA-SMS
Supplementary Figures 1

Supplementary Note 2. Magnetization process of artificial antiferromagnetic Co/Ru/Co tri-layer
Supplementary Figures 2

Supplementary Note 3. Sign reversal of voltage-dependence of H_{ex}
Supplementary Figures 3

Supplementary Note 4. Material design for implementation of VCMA-SMS in devices
Supplementary Figures 4

Supplementary Note 5. Macrospin simulation

Supplementary References

Supplementary Note 1. Measurement setup for voltage-controlled H_{ex} and VCMA-SMS

For the measurement of the voltage-controlled effective H_{ex} and the VCMA-SMS in the microfabricated devices, we used a polar-MOKE (p-MOKE) apparatus equipped with a micro-probe system as shown in Supplementary Fig.1. A semiconductor laser with a wavelength of 408 nm was focused by an objective lens to a spot size of 1.5 μm, which was smaller than the area of the tunnel junctions (10×8 μm). The change in the p-MOKE signal was detected by photodetector in a magnetic field applied perpendicular to the film plane by using an electromagnet. The dc voltage was applied to the samples via a two-terminal rf probe. The dc voltage was generated by a source measure unit (SMU), and the pulse voltage with a width of 10 – 200 ns was generated by an arbitrary function generator (AFG).

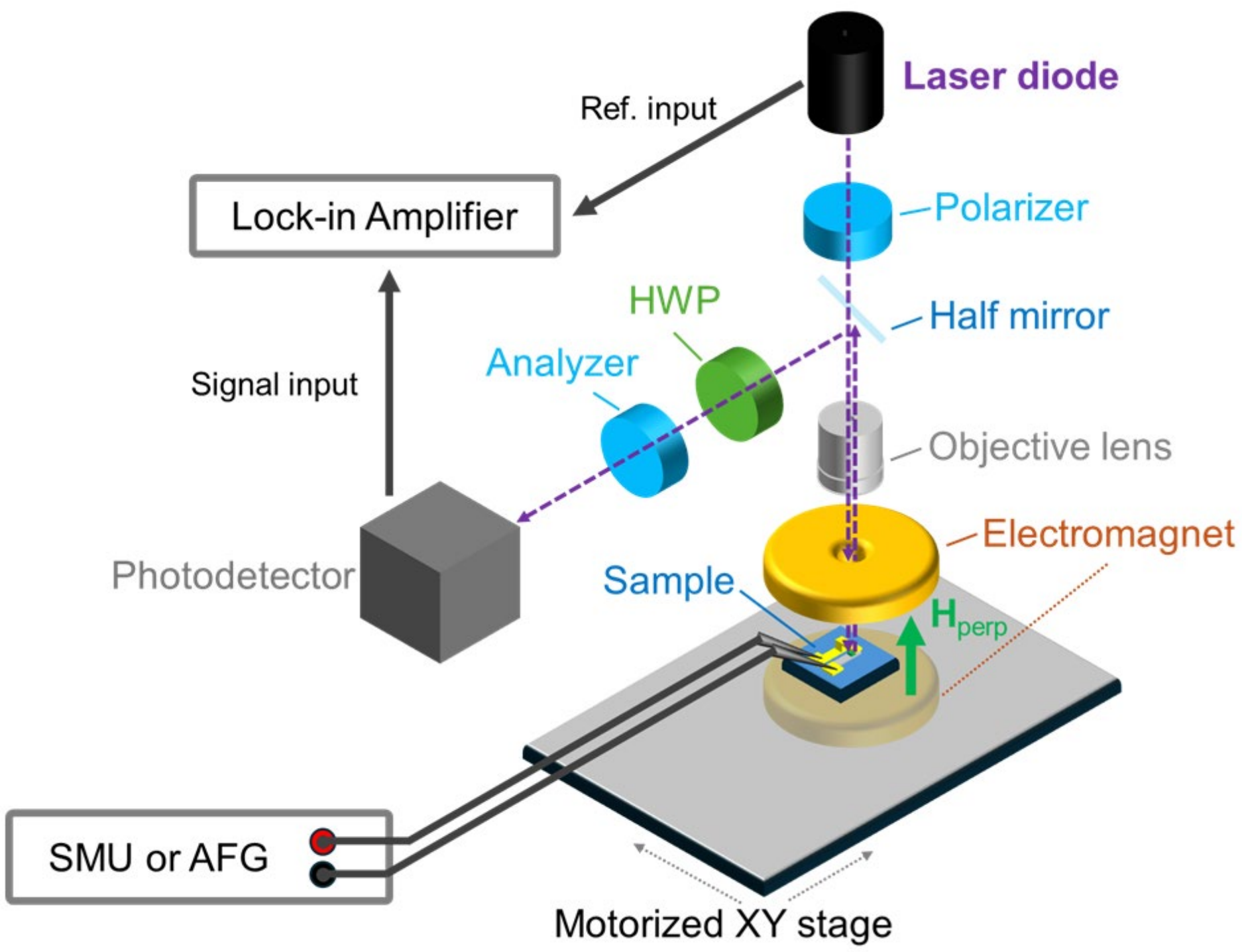

Supplementary Fig.1| Schematic diagram of experimental setup. Voltage-controlled effective H_{ex} and VCMA-SMS were observed by using a polar-MOKE apparatus equipped with micro-probe system.

Supplementary Note 2. Magnetization process of artificial antiferromagnetic Co/Ru/Co tri-layer

Here, we discuss the p-MOKE magnetization curve (Fig.2c) for Co/Ru/Co with t_{Ru}=0.35 nm and t_{Ir}=0.11 nm, which exhibits an antiferromagnetic interlayer exchange coupling (IEC). Supplementary Figure 2 shows the magnetization curve of Fig.2c at a different scale on the y axis to show the magnetization process in more detail. The abrupt changes of p-MOKE signal at $\mu_0 H_{perp}$ = ±1.05 T are due to the switching between AP and nearly P states. In addition to the abrupt changes at $\mu_0 H_{perp}$ = ±1.05 T, the p-MOKE signal abruptly changes at +0.45 T for increasing field and at –0.45 T for decreasing field. For this minor change, the magnetizations of both top and bottom Co layers are reversed while maintaining the AP configuration. This type of reversal occurs because there exists a small but finite uncompensated magnetization of the artificial antiferromagnetic tri-layer, which originates from a slight imbalance of the magnetizations of top and bottom Co layers. This imbalance is reversed by the applied magnetic field. Therefore, $\mu_0 H_{perp}$ = ±0.45 T corresponds to a coercive field of the Co/Ru/Co tri-layer. The small imbalance of the magnetizations is considered to originate from a slightly thicker magnetic dead layer in the top Co layer. It should be noted that we can intentionally imbalance the magnetizations by designing the bottom Co layer thicker than the top Co layer. Note also that the p-MOKE signal drops at the reversal of uncompensated magnetization because the top Co layer contributes to the p-MOKE signal more than the bottom Co layer due to the absorption of light. Thus, the magnetization curve can be divided into five regions, (a) – (e). In (a) and (e), the magnetization configuration is nearly P. In (b) – (d), the magnetization configuration is AP. In (d), the magnetizations of the top (bottom) Co layers are directed downward (upward), which we call AP1 state. In (b), the magnetizations of the top (bottom) Co layers are directed upward (downward), which we call AP2 state. In (c), AP1 and AP2 states are bistable.

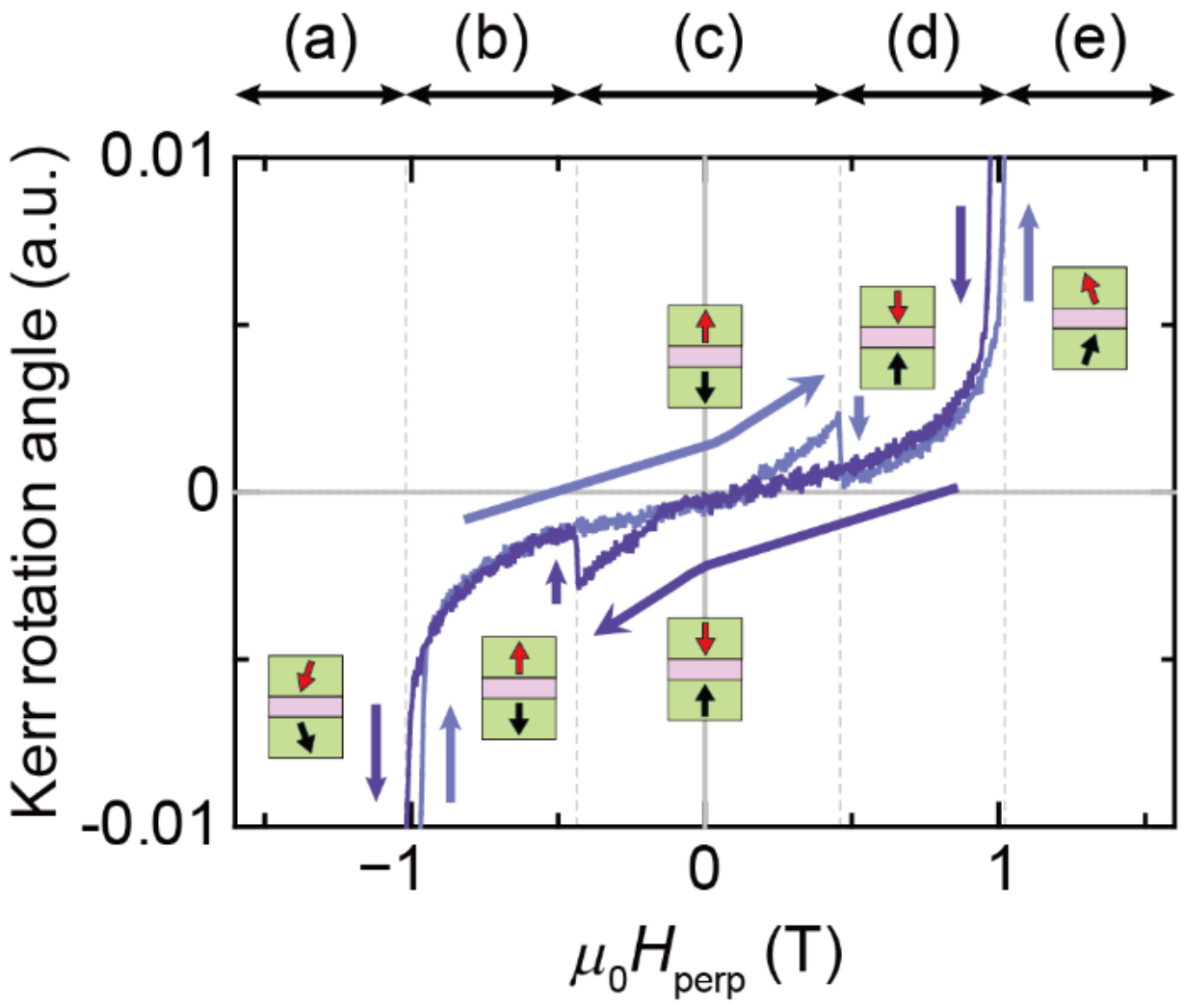

Supplementary Fig.2| Magnetization process of Co/Ru/Co tri-layer. Magnetization curve of Co/Ru/Co tri-layer measured with p-MOKE, where Ru thickness (t_{Ru})=0.35 nm and Ir thickness (t_{Ir})=0.11 nm.

Supplementary Note 3. Sign reversal of voltage-dependence of H_{ex}

The origin of the voltage-controlled effective H_{ex} is further evidenced by changing the sign of the VCMA effect. Although the sign of VCMA in Co/MgO junctions is usually negative, it has been demonstrated that a positive VCMA effect can be obtained by inserting a heavy metal element (typically Pt) followed by post oxidization[1]. In devices with Co/Pt/oxidation process/MgO/ITO stacking structure, the application of positive (negative) voltage increases (decreases) the PMA as illustrated in Supplementary Fig.3a. Therefore, the application of positive (negative) voltage is expected to increase (decrease) H_{ex}, which is opposite to the case of Co/Ru/Co/Ir/MgO/ITO in Fig.3b. For this control experiment, we used Ta(5 nm)/Ru(10 nm)/Ta(5 nm)/Pt(4 nm)/Co(1.1 nm)/Ru-spacer(t_{Ru}=0-0.9 nm)/Co(1.7 nm)/ultrathin-Pt(t_{Pt}=0-0.28 nm)/oxidation process/MgO(3 nm)/ITO(20 nm) polycrystalline film deposited on a thermally oxidized silicon substrate (see Supplementary Fig.3b and Methods). The oxidation process was conducted in a pure oxygen atmosphere at a pressure of 0.5 Pa for 3 minutes. Because the effective thickness of top Co layer is known to decrease by about 0.6 nm due to the oxidation process[1], the designed thickness of the top Co layer is larger than that of the bottom Co layer.

The sign reversal of the voltage-dependence of H_{ex} was investigated using the p-MOKE measurement system. The p-MOKE magnetization curves for t_{Ru}=0.42 nm and t_{Pt}=0.11 nm at positive and negative bias voltages (V_{bias}) are plotted in Supplementary Fig.3c. As expected, the sign of the voltage-dependence of H_{ex} was reversed. As shown in Supplementary Fig.3d, the $\mu_0 H_{ex}$ linearly depends on V_{bias}, which excludes the possibility of charge trapping and chemical reactions in this experiment. The magnitude of $\Delta\mu_0 H_{ex}/V_{bias}$ is smaller than that of unoxidized samples simply because the VCMA effect in the oxidized samples is smaller. The sign of voltage dependence of H_{ex} is clearly reversed when the sign of the VCMA effect is reversed. This is another evidence that the origin of the voltage-controlled effective H_{ex} and J_{ex} is the VCMA effect.

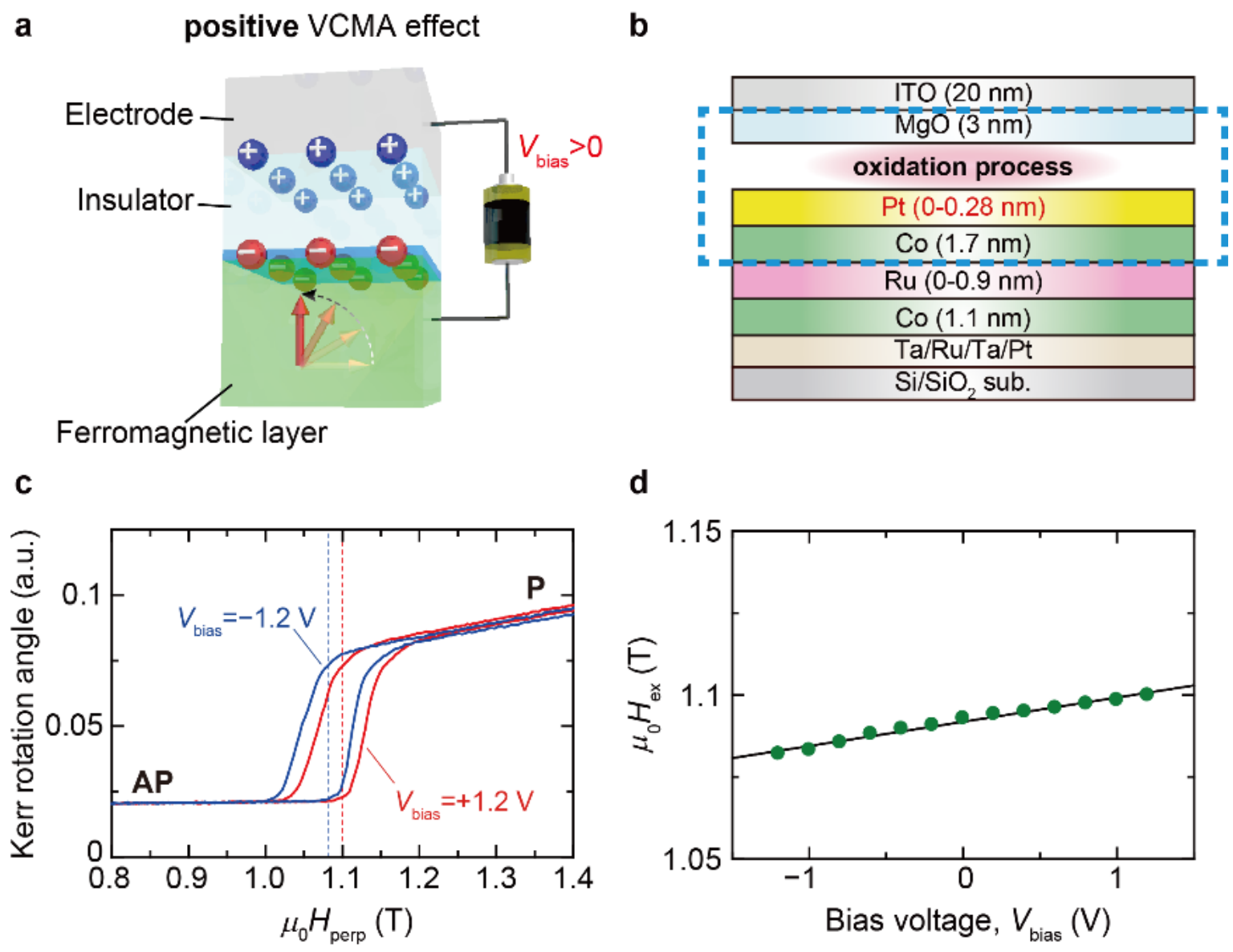

Supplementary Fig.3| Sign reversal of voltage-dependence of H_{ex} by using Co/MgO-based junctions with positive VCMA effect. a, Schematic of the positive VCMA effect. PMA increases with positive V_{bias}. **b,** Schematic of the surface-oxidized Co/Ru/Co multilayer structure used for the control measurements. A positive VCMA effect is obtained by using a Pt insertion layer and conducting an oxidation process[1]. **c,** p-MOKE magnetization curves in vicinity of $\mu_0 H_{ex}$, where t_{Ru}=0.42 nm and t_{Pt}=0.11 nm. **d,** $\mu_0 H_{ex}$ plotted as function of V_{bias}.

Supplementary Note 4. Material design for implementation of VCMA-SMS in devices

In this section, we discuss an example of a multilayer structure for implementation in actual devices. For implementation, the multilayer structure should possess a sufficiently large tunneling magnetoresistance (TMR) effect and be capable of voltage induced magnetization switching under low magnetic fields. Supplementary Figure 4 shows a schematic of the multilayer structure, which appears to be feasible for implementation. By using a relatively thick single pinned layer, it is possible to generate an offset magnetic field of approximately 0.1 T, which is expected to enable the application of an effective magnetic field without incorporating isolated nano magnets. The stacking structure on the MgO tunnel barrier can be deposited by a sputtering process at cryogenic temperature[2,3]. It has been demonstrated that boron-free CoFe-based ultrathin films deposited on MgO at cryogenic temperature exhibit sufficiently high VCMA coefficient (~200 fJ/Vm)[4]. By doping CoFe-based alloys with heavy metals such as Ir, Pt, and Pd to increase both the PMA and Gilbert damping, the magnetization switching due to the VCMA-SMS is expected to be faster, and the retention is expected to be longer. Although doping heavy metals into CoFe-based alloys is known to reduce the TMR effect, an ultra-thin Co(Fe) termination layer introduced between Ir-doped CoFe and MgO tunnel barrier can recover TMR. It should be noted that Ir atoms doped into the second interfacial atomic layer of the ferromagnetic layer are known to enhance both the VCMA effect and the PMA[5]. Therefore, both a large VCMA coefficient and a relatively high MR can be achieved by introducing an ultra-thin Co(Fe) termination layer between the Ir-doped CoFe and the MgO[6].

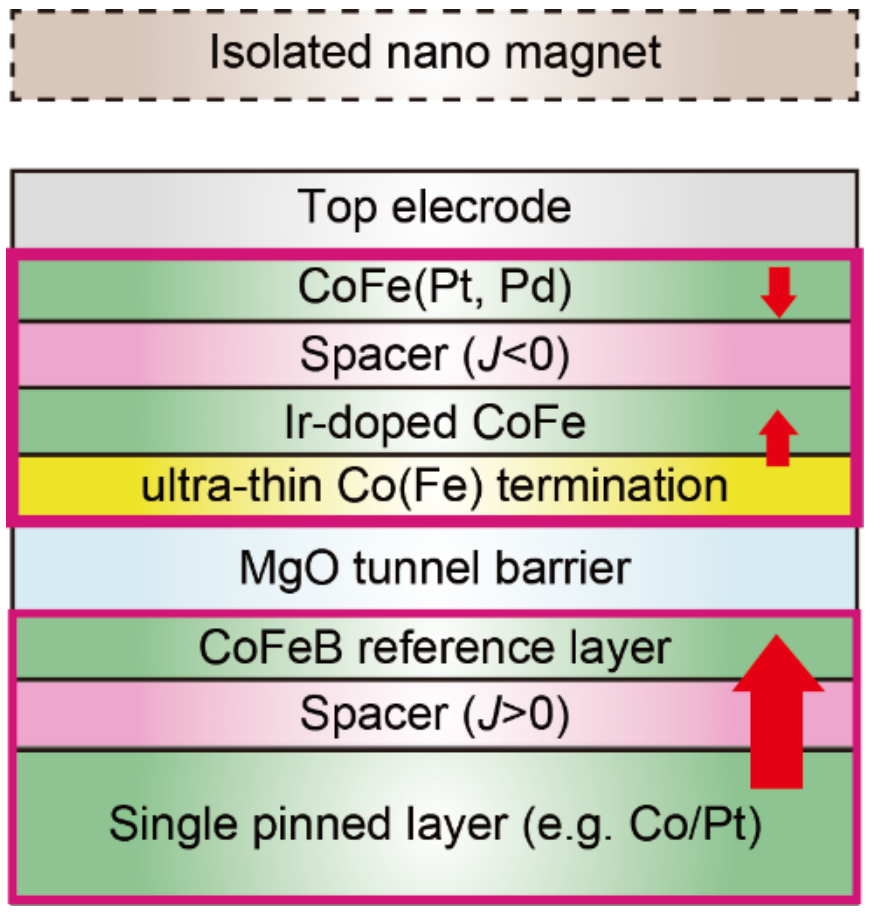

Supplementary Fig.4| Material and design for implementation of VCMA-SMS in a magnetic tunnel junction. Schematic of a possible stacking structure for implementation in devices. By using a single pinned layer, it is possible to generate an offset magnetic field without incorporating isolated nano magnets.

Supplementary Note 5. Macrospin simulation

In the macrospin simulations, the magnetization dynamics and equilibrium states were determined by solving the following Landau-Lifshitz-Gilbert (LLG) equation:

$$\frac{d\boldsymbol{m}_i}{dt} = -\gamma\, \boldsymbol{m}_i \times \boldsymbol{H}_i + \alpha_i\, \boldsymbol{m}_i \times \frac{d\boldsymbol{m}_i}{dt},$$

where γ is the gyromagnetic ratio, $\boldsymbol{m}_i$ is the magnetization unit vector, $\boldsymbol{H}_i$ is the effective field, and α_i is the Gilbert damping constant. The subscript $i =$ 1 and 2 for the top and bottom magnetic layers, respectively. The effective field is composed of the perpendicular external magnetic field ($\boldsymbol{H}_{\mathrm{perp}}$), the anisotropy field ($\boldsymbol{H}_{K,i}$), and the exchange field ($\boldsymbol{H}_{J,i}$), as expressed by

$$\boldsymbol{H}_i = H_{\mathrm{perp}}\boldsymbol{e}_z + \boldsymbol{H}_{K,i} + \boldsymbol{H}_{J,i},$$

where

$$\boldsymbol{H}_{K,i} = \frac{2K_i}{\mu_0 M_{s,i}} m_{z,i}\boldsymbol{e}_z$$

and

$$\boldsymbol{H}_{J,i} = \frac{J}{\mu_0 M_{s,i} t_i}\boldsymbol{m}_{(j\neq i)}.$$

Here J is the exchange coupling constant between the top and bottom ferromagnetic layers, μ_0 is the permeability of vacuum, and t_i is the thickness of the ith layer.

The multilayer structure used for the calculations of the voltage-controlled effective exchange field H_{ex} is an F2/N-spacer/F1 tri-layer. The thicknesses of the first and second layers, t_1 and t_2, were assumed to be equal to 1.0 nm. The saturation magnetization of the F1 and F2 $M_{\mathrm{S},1}$ and $M_{\mathrm{S},2}$ were assumed to be equal to 1400 kA/m. The anisotropy constant of the F1 layer was assumed to be K_1=0.22×10^6 J/m^3 at an applied voltage of +1.2 V and 0.135×10^6 J/m^3 at −1.2 V. The anisotropy constant of F2 layer was assumed to be K_2=0.185×10^6 J/m^3 regardless of the applied voltage. The exchange constat was assumed to be J=−3.25×10^{-3} J/m^2 regardless of the applied voltage. The LLG equation was numerically solved by using Mathematica.

In the calculation of the magnetization curve shown in Fig. 3e, the Gilbert damping constants were set to α_1= α_2=1.0 to reduce the computation time of the simulation. In the calculation of the switching time, realistic values of the Gilbert damping constants, α_1= α_2=0.05, were assumed.

Supplementary References

1. Nakayama, H., Nozaki, T., Nozaki, T. & Yuasa, S. Engineering of Co/MgO interface with combination of ultrathin heavy metal insertion and post-oxidation for voltage-controlled magnetic anisotropy effect. *APL Mater* **12**, 091122 (2024).
2. Ichinose, T. *et al.* Cryogenic Temperature Deposition of High-Performance CoFeB/MgO/CoFeB Magnetic Tunnel Junctions on φ300 mm Wafers. *ACS Appl Electron Mater* **5**, 2178–2183 (2023).
3. Sugihara, A. *et al.* Low magnetic damping in an ultrathin CoFeB layer deposited on a 300 mm diameter wafer at cryogenic temperature. *Applied Physics Express* **16**, 023003 (2023).
4. Ichinose, T. *et al.* Cryogenic-temperature grain-to-grain epitaxial growth of high-quality ultrathin CoFe layer on MgFeO tunnel barrier for high-performance magnetic tunnel junctions. *NPG Asia Mater* **17**, 41 (2025).
5. Nozaki, T. *et al.* Highly efficient voltage control of spin and enhanced interfacial perpendicular magnetic anisotropy in iridium-doped Fe/MgO magnetic tunnel junctions. *NPG Asia Mater* **9**, e451 (2017).
6. Nozaki, T. *et al.* Voltage-controlled magnetic anisotropy in an ultrathin Ir-doped Fe layer with a CoFe termination layer. *APL Mater* **8**, 011108 (2020).
